# Influence of *Panax ginseng* on the offspring of adult rats exposed to prenatal stress

**DOI:** 10.3892/ijmm.2014.2003

**Published:** 2014-11-13

**Authors:** YOUNG OCK KIM, HWA-YOUNG LEE, HANSOL WON, SEONG-SU NAH, HWA-YOUNG LEE, HYUNG-KI KIM, JUN-TACK KWON, HAK-JAE KIM

**Affiliations:** 1 Development of Ginseng and Medical Plants Research Institute, Rural Administration, Eumseong, Republic of Korea; 2 Department of Clinical Pharmacology, College of Medicine, Soonchunhyang University, Cheonan, Republic of Korea; 3 Division of Rheumatology, Department of Internal Medicine, College of Medicine, Soonchunhyang University, Cheonan, Republic of Korea; 4 Department of Psychiatry, College of Medicine, Soonchunhyang University, Cheonan, Republic of Korea; 5 Soonchunhyang Medical Research Institute, College of Medicine, Soonchunhyang University, Cheonan, Republic of Korea

**Keywords:** *Panax ginseng*, prenatal stress, social interaction

## Abstract

The exposure of pregnant females to stress during a critical period of fetal brain development is an environmental risk factor for the development of schizophrenia in adult offspring. Schizophrenia is a group of common mental disorders of unclear origin, affecting approximately 1% of the global population, showing a generally young age at onset. In the present study, a repeated variable stress paradigm was applied to pregnant rats during the final week of gestation. The effects of an extract of *Panax ginseng* C.A. Meyer (PG) on rats exposed to prenatal stress (PNS) were investigated in terms of behavioral activity and protein expression analyses. In the behavioral tests, grooming behavior in a social interaction test, line-crossing behavior in an open-field test and swimming activity in a forced-swim test were decreased in the rats exposed to PNS compared with the non-stressed offspring; the changes in behavioral activity were reversed upon oral treatment with PG (300 mg/kg). Subsequently, western blot analysis and immunohistochemical analyses of the prefrontal cortex and hippocampus revealed that the downregulation of several neurodevelopmental genes which occurred following exposure to PNS was reversed upon treatment with PG. The current findings demonstrate that the downregulation of several genes following exposure to PNS may affect subsequent behavioral changes, and that these phenomena are reversed following treatment with PG during pregnancy. Our results suggest that oral treatment with PG reduces the incidence of psychiatric disorders, such as schizophrenia.

## Introduction

Prenatal stress (PNS) is an important environmental risk factor for the development of schizophrenia in adults, and the second trimester of pregnancy in humans seems to be the most vulnerable period for insults (1–6). Additionally, various animal studies have demonstrated that PNS during gestation elevates glucocorticoid levels and is related to biochemical, physiological and behavioral changes in the offspring, including reduced birth weight, cardiovascular and neuroendocrinological abnormalities, attentional dysfunction, enhanced anxiety-related behaviors and cognitive deficits (7–16). For the study of PNS, pregnant female rats are commonly exposed to stressful manipulations during the third week of pregnancy, which, in terms of neural development, is approximately equivalent to the second trimester of human gestation (13–15).

In animal studies, exposure to PNS has been shown to result in a diminished number of hippocampal synapses and fewer neurons in the brain (17). Moreover, the hippocampi of adolescent and adult male rats exposed to PNS exhibit decreases in dendritic length, spine density and the number of neurons relative to non-stressed (NS) controls (18,19). PNS has also been shown to cause variable changes in gene expression in the rat brain, including the expression of genes associated with neuronal development, cell differentiation and neurotransmitter function (14,20,21). As shown in our previous study, dihydropyrimidinase like protein (Dpysl)2 and 3 were downregulated in the prefrontal cortex of PNS rats and polymorphisms in the *DPYSL2* gene in humans may be associated with the development of schizophrenia (22).

Ginseng, the root of *Panax ginseng* C.A. Meyer (PG), has been used traditionally as an herbal medicine for over 1,000 years in East Asian countries, such as China, Japan and Korea (23). PG is effective in the treatment of various disorders, such as shallow respiration, shortness of breath, cold limbs, lack of appetite, chest and abdominal distension and profuse sweating (24). Researchers have focused on the novel pharmacological effects of PG; some have attempted to explain the pharmacological aspects of PG in various abnormalities in humans, such as cancer, diabetes mellitus and neurodegenerative disorders (25,26). PG has also been widely used to enhance stamina and to address fatigue and physical stress for thousands of years in Traditional Oriental medicine (27). A recent placebo-controlled study indicated that the verbal working memory and visual working memory of patients with schizophrenia was improved by treatment with a ginseng extract (28).

The abovementioned findings have resulted in the hypothesis that PG may affect the pathophysiology of schizophrenia. Thus, in the present study, the behavioral patterns in the offspring and changes in protein levels were examined in the prefrontal cortex and hippocampus of rats exposed to PNS, and whether treatment with PG reverses these changes which occur due to PNS.

## Materials and methods

Dried roots of PG were purchased from Yunpung, Chungbuk, Korea, and the specimens were identified taxonomically by an Oriental medicine physician at the National Institute of Horticultural and Herbal Science, Rural Development Administration (RDA), Suwon, Korea. The voucher specimen (HPR-207) was deposited at the herbarium of the Herbal Crop Research Institute (Eumsung, Korea).

We used water extraction as the majority of traditional Oriental herbal materials have been decocted with boiling water, and as ginsenosides are more soluble in water than in organic solvents. The crushed plant materials (200 g each) were extracted under reflux with distilled water 3 times. The combined water extracts were lyophilized and the yield was 18.3% (wt/wt) for PG in the dried state. They were stored at −20°C until use. Powdered extracts of PG were dissolved in saline to a concentration of 300 mg/ml. The animals were orally administered PG solution during pregnancy.

The rat model of PNS was prepared as described in previous studies with slight modifications (13,14). Pregnant Sprague-Dawley rats were purchased from Central Laboratory Animal Inc. (Seoul, Korea) and arrived at the animal facility on day 7 of gestation. The rats were housed under standard conditions with a 12/12-h light/dark cycle (lights on at 06:30) with free access to food and water. All animal procedures were performed in accordance with the guidelines for the care and use of laboratory animals of the US National Institutes of Health.

Beginning on day 14 of gestation, exposure to PNS was initiated and consisted of: i) restraint in well-ventilated cylindrical plexiglas restrainers for 1 h; ii) exposure to a cold environment (4°C) for 6 h; iii) overnight food deprivation; iv) 15 min of swim stress in room-temperature water; v) reversal of the light-dark cycle; and/or vi) social stress induced by overcrowded housing conditions during the dark phase of the cycle (13,14). Pregnant rats used as the controls remained in the animal room during gestational days 14–21 and were exposed to only normal animal-room husbandry procedures.

Following birth, the rats and their pups were left undisturbed in their cages until weaning on postnatal day 23. At this time, the male and female offspring were separated and group-housed in cages with 1 or 2 littermates of the same gender with free access to rat chow and water. The animals were exposed to normal animal room conditions from that point forward until experimental use on postnatal day 35 (14,15).

Modified behavioral tests, including a social interaction test, the open-field test (OFT) and the forced-swim test (FST), were carried out as previously described (13,29–31). The social interaction test was adapted from previous studies and was conducted in a clear plexiglas chamber (77×77×25 cm) (13,30,31). The room in which the chamber was located was darkened during testing, and the chamber was illuminated by a single 25 W red light bulb placed ~100 cm above the base of the chamber (subject age, 30 days). Sessions were filmed with a video camera placed 150 cm above the cage. The experimenter remained outside the test room during testing, and the test arena was cleaned after each session. Social interaction partners were siblings of the same gender who resided in the same cage after weaning and were of approximately equal body weight (in the few cases where a sibling of the same gender was not available, a playmate from similar conditions was used). Each session lasted for 20 min and was scored in terms of the total duration of social play and the numbers and types of interactions. Specifically, a rater blinded to the treatment conditions scored behavioral activity as aggressive [fighting (kicking, boxing and wrestling), aggressive grooming and biting] or non-aggressive (sniffing, following and grooming the partner) based on the video. Experimental and target rats were not used in this paradigm more than once, and the arena was cleaned with 70% ethanol after each trial. The OFT was used to assess exploratory activity and reactivity to a novel environment. On the test day, the subjects were removed from their home cage (subject age, 32 days) and were placed individually in the start box (15×15×20 cm) of the open field arena (77×77×25 cm) for 5 min. The apparatus was composed of black Polygal and no background noise was provided. The experimenter exited the room, and the behavior of the subject was recorded. Scoring included central boxes entered, line crossings, runs, rears, grooming, cage sniffs and immobile behavior, as previously described (29,30).

The modified FST was used (subject age, 34 days), as described in previous studies (29,30). The rats were lowered individually into a cylinder (height, 40 cm; diameter, 20 cm) filled with fresh, warmed tap water (25±2°C). After 5 min, the rats were removed and wiped with a clean towel to remove excess water before being returned to their home cage. On the following day, each rat was again placed in the cylinder for 15 min during which the time spent swimming and climbing the time spent immobile were recorded with a video camera and by an observer using a stopwatch. The predominant behaviors were counted every 5 sec. Test scores were recorded and included swiming activity (horizontal movement throughout the chamber and crossing quadrants), climbing activity (upward-directed movements up the side of the chamber and jump-ups from the bottom of the chamber) and immobility (no additional activity other than keeping the head above water or tiny whip kicks, as previously described by Dulawa *et al* (29) and Schroeder *et al* (30).

The rats were anesthetized deeply with ethyl ether and perfused with 4% paraformaldehyde. The fixed brains were removed, frozen and cut into 30-μm sections. To detect Dpysl2 expression and neurofilament expression, frozen sections from the rat prefrontal cortex and hippocampus were blocked with normal horse serum, incubated with anti-Dpysl2 antibody (1:200; Cell Signaling Technology, Danvers, MA, USA), NF200 antibody (1:160; Sigma, St. Louis, MO, USA) or NeuN (1:100; EMD Millipore, Billerica, MA, USA) and then incubated with a Cy3-conjugated anti-rabbit (Dpysl2) and mouse (NF200) and a FITC-conjugated anti-mouse (NeuN) secondary antibody secondary antibody (1:200, 1:500 and 1:500; Jackson ImmunoResearch Laboratories, Inc., West Grove, PA, USA). Fluoroshield™ with DAPI (Sigma) was used for the nuclear staining of the brain tissues and mounting of the slides. Fluorescence images were captured using a confocal laser-scanning microscope (FV10-ASW; Olympus, Tokyo, Japan), and image quantification was performed with ImageJ software using a protocol previously described with slight modifications (33).

Prefrontal cortical and hippocampal tissues were lysed in radioimmunoprecipitation assay (RIPA) buffer containing protease inhibitors and then centrifuged at 14,000 rpm for 10 min at 4°C. To identify Dpysl2 and neurofilament protein, 100 μg of the lysed protein were placed on a 10 and 8% SDS gel and transferred onto a polyvinylidene difluoride (PVDF) membranes (EMD Millipore). After blocking with 5% skim milk, the membranes were probed with anti-Dpysl2 (1:1,000; no. 9393; Cell Signaling Technology, Inc.), anti-NF200 (1:1,000; N4142; Sigma), or anti-β-actin (Actb; 1:1,000; sc-81178; Santa Cruz Biotechnology, Inc., Santa Cruz, CA, USA) antibodies overnight at 4°C and then with peroxidase-conjugated secondary antibody (1:10,000; Sigma) for 1 h at room temperature. Immunoreactive bands were detected using an enhanced chemiluminescence kit (Elpis Biotech Inc., Daejeon, Korea), and quantitative measurements of Dpysl2, NF200 and the Actb protein were obtained using ImageJ software.

Difference between groups were analyzed using the Student’s t-test. P-values <0.05 were considered to indicate a statistically significant difference.

## Results

To evaluate the extent to which treatment with PG alters behavioral activity and protein expression changes that may be related to the pathophysiology of schizophrenia during maternal stress in pregnancy, we used a model of variable and unpredictable stress in rats. Pregnant females were exposed to various stressors from day 14 to 21 of gestation. We investigated the effects of treatment with PG on the PNS-induced behavioral phenotypes using an OFT, FST and a social interaction test.

We observed significant differences among the control, PNS and PG-treated groups in the social interaction test (Table I). In particular, one of the non-aggressive behaviors, the number and duration of sniffs, was decreased significantly in the PNS group (P<0.05; Table I) and was restored by oral treatment with PG.

**Figure 1:**
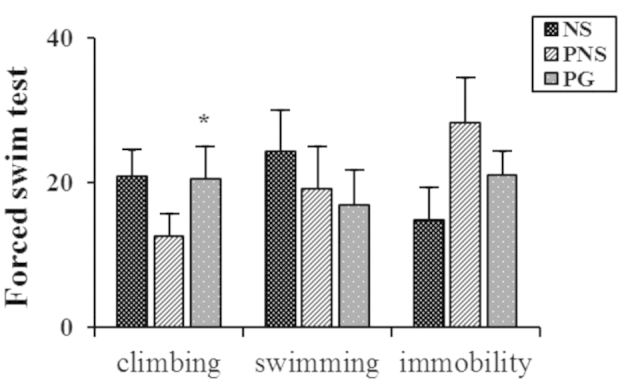
Behavioral response in the forced-swim test. Comparison between offspring of the NS and PNS groups (n=10 in each group). A decrease in swimming activity was observed. Data are presented as the means ± SEM. ^*^P<0.05. NS, offspring of non-stressed rats; PNS, offspring of rats exposed to prenatal stress; PG, rats exposed to PNS and treated with *Panax ginseng* extract.

In the FST, the offspring of the rats exposed to PNS exhibited decreased climbing activity and increased immobility compared with the non-stressed (NS) rat offspring (P<0.05; Fig. 1). The climbing activity was recovered upon treatment with PG (P<0.05; Fig. 1).

The offspring of the NS rats and those exposed to PNS were tested in the open field for 20 min. The PNS group showed a significantly decreased number of central entries and line crossings; these scores recovered following treatment with PG (P<0.05; Table II). In addition, the PNS group showed a significantly decreased number and duration of runs and rear behavior; these scores recovered upon treatment with PG (P<0.05; Table II). Similarly, the number of times immobility occurred and the duration of the immobility increased in the PNS group and returned to the control levels upon treatment with PG (P<0.05; Table II).

**Figure 2:**
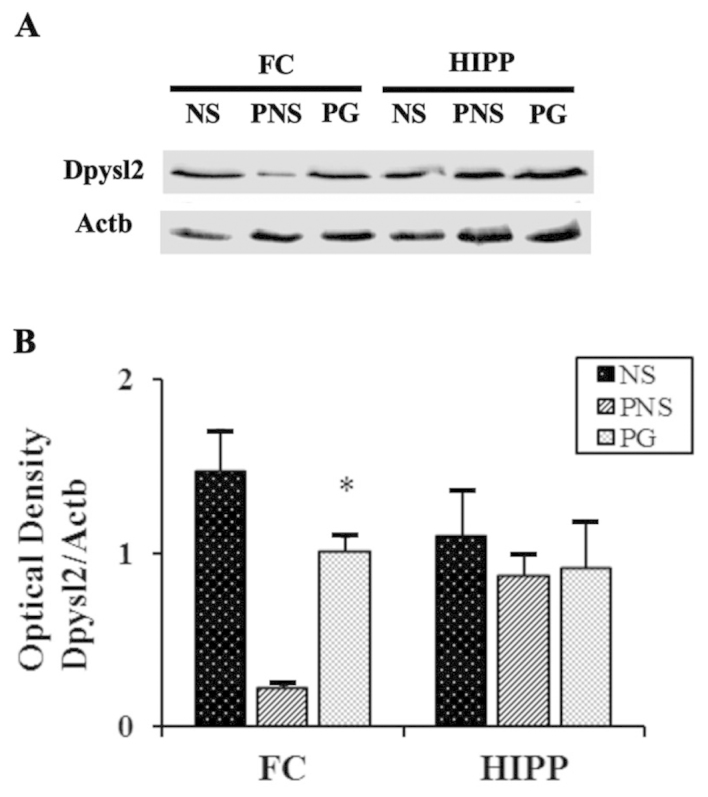
Western blot analysis of dihydropyrimidinase like protein (Dpysl)2 expression. (A) Dpysl2 expression was detected by western blot anlaysis with anti-β-actin antibody (Actb) as an internal control. Rats exposed to prenatal stress (PNS) exhibited decreased Dpysl2 expression in the prefrontal cortex and hippocampus. (B) Quantitative analysis of western blot anlaysis data for Dpysl2 expression showed a significant difference in Dpysl2 levels in the PNS vs. the PG-treated group (^*^P<0.05 compared with the NS group in the prefrontal cortex and hippocampus). FC, prefrontal cortex; HIPP, hippocampus; NS, offspring of non-stressed rats; PNS, offspring of rats exposed to prenatal stress; PG, rats exposed to PNS and treated with *Panax ginseng* extract.

**Figure 3:**
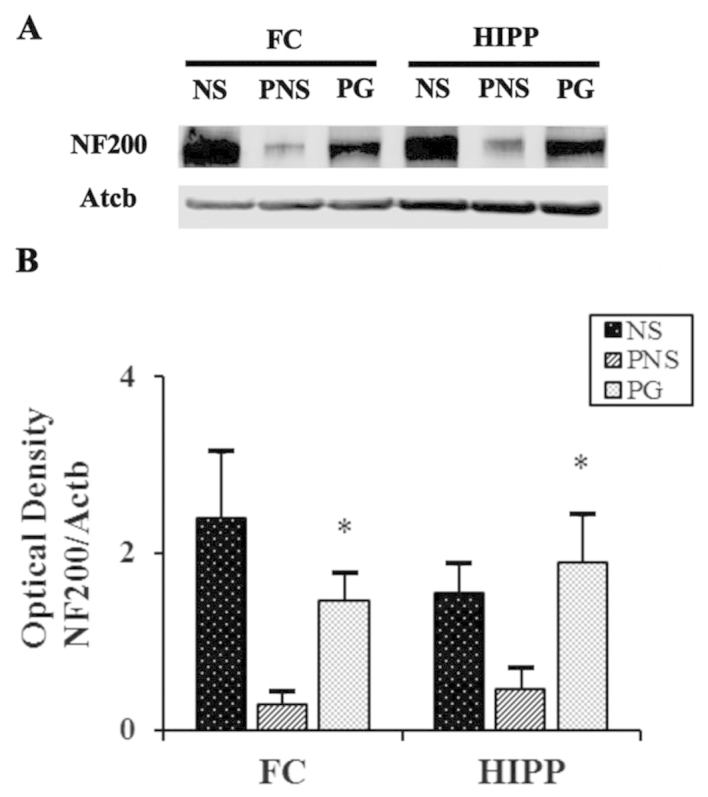
Western blot analysis of neurofilament protein (NF200) expression. (A) NF200 expression was detected by western blot analysis with anti-β-actin antibody (Actb) as an internal control. Rats exposed to prenatal stress (PNS) exhibited decreased NF200 expression in the prefrontal cortex and hippocampus. (B) Quantitative analysis of western blot analyiss data for NF200 expression showed a significant difference in NF200 levels in the PNS vs. the PG-treated group (^*^P<0.05). FC, prefrontal cortex; HIPP, hippocampus; NS, offspring of non-stressed rats; PNS, offspring of rats exposed to prenatal stress; PG, rats exposed to PNS and treated with *Panax ginseng* extract.

**Figure 4:**
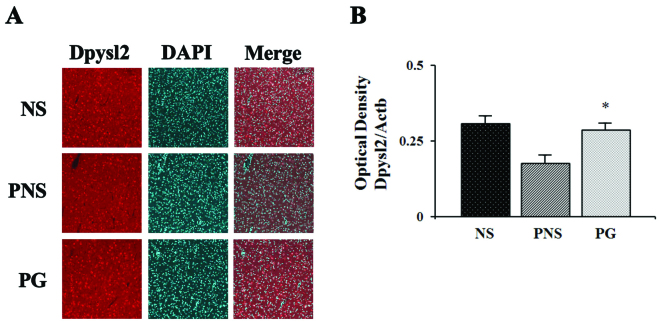
Immunohistochemical analysis of dihydropyrimidinase like protein (Dpysl)2 expression. (A) Confocal microscopy image showing immunofluorescent staining for Dpysl2 (anti-Dpysl2, red, Cy3) with DAPI (blue) in the prefrontal cortex. Scale bar, 50 μm. (B) Optical densities of Dpysl2 signals in immunostained prefrontal cortex sections (^*^P<0.05 compared with the NS group in the prefrontal cortex and hippocampus). FC, prefrontal cortex; NS, offspring of non-stressed rats; PNS, offspring of rats exposed to prenatal stress; PG, rats exposed to PNS and treated with *Panax ginsen*g extract.

**Figure 5:**
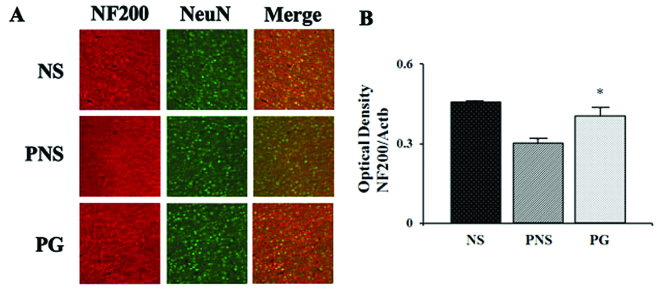
Immunohistochemical analysis of neurofilament protein (NF200) expression. (A) Confocal microscopy image showing immunofluorescence staining for Dpysl2 (anti-NF200, red, Cy3) and neuronal nuclei (anti-NeuN, green and FITC) in the prefrontal cortex. Scale bar, 50 μm. (B) Optical densities of NF200 signals in immunostained prefrontal cortex sections (^*^P<0.05). FC, prefrontal cortex; NS, offspring of non-stressed rats; PNS, offspring of rats exposed to prenatal stress; PG, rats exposed to PNS and treated with *Panax ginseng* extract.

Dpysl2 is expressed in neurons of the central nervous system and is concentrated at synaptic sites and in the axons, where it may affect synaptic physiology (33). In our previous study, we demonstrated that Dpysl2 was decreased in the offspring of rats exposed to PNS (22). To investigate the PNS-induced downregulation of Dpysl2 protein and the effects of PG treatment, we performed western blot analysis (Fig. 2) and immunohistochemical anlaysis (Fig. 3) of the prefrontal cortex areas of the brains of rats in the control, PNS and PG group. Western blot analyses revealed that the expression levels of Dpysl2 and neurofilament proteins in the prefrontal cortex were significantly lower in the rats exposed to PNS than in the control rats (all P-values <0.05; Figs. 2 and 3), whereas the expression levels of of Actb in the prefrontal cortex were similar in all groups. This differential expression of Dpysl2 and that of neurofilament proteins was also evident in the immunofluorescence-stained images of the brains of rats in the control, PNS and PG group and in the measurements of immunohistochemical staining intensity (all P-values <0.05; Figs. 4 and 5).

## Discussion

In the present study, to examine the effects of PG on the pathophysiology of stress-related psychiatric disorders, such as schizophrenia, according to the neurodevelopmental theory, we performed behavioral and protein expressional analyses in an animal model of PNS.

Ginseng has been used in Asia for thousands of years to improve vitality, wakefulness, respiration, angina, nausea, attention span, memory, immune function and diminished libido, and has more recently become one of the most popular herbal supplements in the Western world (23,34–36). Technological advances have led to the identification, characterization and standardization of the active components in ginseng extracts. Ginsenosides are unique triterpenoid saponins found exclusively in PG, and to date more than 150 naturally occurring ginsenosides have been isolated from ginseng (37–39). The biological funcionts of PG are complex and include some effects that may be related to mental disorders, such as affective and anxiety disorders, through the modulation of the hypothalamic-pituitary-adrenal (HPA) axis and the monoaminergic system (40,41).

In the context of novel theories related to depression, the active ingredients of PG have been demonstrated to exert neuroprotective effects and to increase neuronal survival. *In vitro*, treatment with ginsenosides has been shown to increase survival and promote neuronal plasticity and neurogenesis in dopaminergic cells (42). *In vivo*, ginsenosides have been shown to reduce hypoxic brain injury in rats, and to protect against toxic interventions in Parkinson’s disease (43–45). A previous study demonstrated the antidepressant effects of total ginseng saponin, which contains several ingredients (46). However, it is important to identify the active ingredient(s) that improve the depression-like behavior. Rg1 has a molecular structure similar to that of ginsenoside Rb3, which possesses antidepressant properties, and has been reported to increase brain-derived neurotrophic factor (BDNF) expression following focal cerebral ischemia (47,48).

The present study provides direct evidence that an extract of PG can significantly recover PNS-induced psychiatric effects in an animal model. Although some prenatal manipulations in rats, such as immune challenge, viral infection and protein malnutrition, also recapitulate sensory gating abnormalities and cognitive disturbances, only unpredictable PNS, hippocampal lesioning and prenatal immune challenge generate social impairment in mice (49–51). Impaired social interaction behavior was observed in rats exposed to PNS rats at 35 days of age, as well as in young adult rats exposed to PNS. One of the first clinical signs associated with human schizophrenia is social withdrawal during adolescence (52–54). The emergence of social withdrawal in adolescent rats exposed to PNS appears to be consistent with the clinical schizophrenia literature and further supports the relevance of this model to the schizophrenia phenotype. This diminution in social interaction behaviors may reflect an increase in anxiety in rats epxosed to PNS (55). In our study, the PNS-induced decrease in non-aggressive behavior was restored by oral treatment with PG. In addition, some behavioral patterns from FST and OFT, tests for the analysis of depressive behaviors, were recovered by treatment with PG. In the present study, we investigated the levels of Dpysl2 and neurofilament proteins. In the developing brain, Dpysl2 regulates axonal outgrowth by promoting microtubule assembly, vesicle trafficking and synaptic physiology (56–59). The expression of *DPYSL2* in humans has been reported to be decreased in the brains of patients with schizophrenia (60). Neurofilaments form part of the axon skeleton and functionally maintain neuronal caliber. They may also play a role in intracellular transport to axons and dendrites (61). These findings suggest that the application of a repeated variable PNS paradigm during the critical periods of fetal brain development results in changes the expression of neurodevelopmental proteins, such as neurofilament proteins and Dpysl2, that may have enduring effects on axonal outgrowth and synaptic function in the offspring later, during adulthood. In the present study, the decrease in the expression of neurofilament proteins and Dpysl2 following epxosure to PNS was shown to be reversed by treatment with PG. PNS during gestation has been implicated in the pathology of various psychiatric disorders, such as schizophrenia and depression.

The present study provides valuable data regarding an additional role of PG in addressing the pathogenesis of psychiatric disorders, such as schizophrenia. However, further research using cellular and animal model systems is required to fully characterize the pharmacological functions of PG.

## Figures and Tables

**Table I:** Analysis of behavior of rats in a social interaction test and effects of PG.

Behavior	Control	PNS	PG
Sniff (n)^a^,^b^	38.43±3.32	24.14±2.21	43.00±4.78
Sniff (s)^a^,^b^	82.57±14.82	37.43±5.82	105.71±17.84
Follow (n)	12.57±3.47	9.00±2.30	4.29±1.63
Follow (s)	22.71±5.92	29.57±17.97	9.29±3.25
Grooming partner (n)	4.43±0.95	2.43±0.72	3.29±0.72
Grooming partner (s)	12.86±3.44	17.57±6.01	14.71±8.59
Fight (n)	2.71±2.23	2.43±1.25	1.43±0.57
Fight (s)	5.86±3.99	4.43±2.22	3.14±1.49
Aggressive (n)	6.00±1.83	3.86±1.42	2.14±0.86
Aggressive (s)	13.57±4.95	20.29±8.02	13.43±8.90
Biting (n)	0.57±0.37	0.29±0.29	0.00±0.00
Biting (s)	0.57±0.37	0.86±0.86	0.00±0.00

Data are presented as the means ± SEM; n, number of times the behavior was observed; s, duration of behaviour measured in seconds; NS, non-stressed group; PNS, prenatally-stressed group; PG, oral administration of PNS group with *Panax ginseng*;

a comparison between the NS and PNS group (P-value <0.05);

b comparison between the PNS and PG group (P-value <0.05).

**Table II:** Behavior of rats in an open-field test and effects of PG.

Behavior	Control	PNS	PG
Central entered^a^,^b^	11.57±3.18	3.43±0.84	17.29±1.91
Line crossing^a^,^b^	3.43±1.25	0.00±.00	4.86±0.80
Run (n)^b^	15.14±7.51	2.29±0.61	12.57±3.39
Run (s)^b^	22.43±13.58	3.14±0.91	15.57±5.26
Rear (n)^b^	77.29±18.97	33.86±9.08	95.00±6.67
Rear (s)^a^,^b^	194.29±28.77	59.14±15.38	203.00±19.26
Grooming (n)	21.00±2.74	14.43±3.80	18.43±2.50
Grooming (s)	241.71±56.06	199.86±42.57	241.14±25.80
Cage sniff (n)	33.43±6.50	31.00±7.28	49.86±6.54
Cage sniff (s)	129.71±21.60	170.86±37.31	148.57±23.76
Immobile (n)^a^,^b^	1.14±0.34	12.43±4.91	0.86±0.40
Immobile (s)^a^,^b^	3.43±1.60	71.43±26.51	2.00±1.23

Data are presented as the means ± SEM; n, number of times the behavior was observed; s, duration of behavior measured in seconds; NS, non-stressed group; PNS, prenatally-stressed group; PG, oral administration of PNS group with *Panax ginseng*;

a comparison between the NS and PNS group (P-value <0.05);

b comparison between the PNS and PG group (P-value <0.05).

## References

[1] Brown AS1, van Os JC (2000). Further evidence of relation between prenatal famine and major affective disorder. Am J Psychiatry.

[2] Sullivan PF (2005). The genetics of schizophrenia. PLoS Med.

[3] Huttunen MO, Niskanen P (1978). Prenatal loss of father and psychiatric disorder. Arch Gen Psychiatry.

[4] King M, Nazroo J, Weich S (2005). Psychotic symptoms in the general population of England: a comparison of ethnic groups (The EMPIRIC study). Soc Psychiatry Psychiatr Epidemiol.

[5] King S, Laplante D, Joober R (2005). Understanding putative risk factors for schizophrenia: retrospective and prospective studies. J Psychiatry Neurosci.

[6] Lim C, Chong SA, Keefe R (2009). Psychosocial factors in the neurobiology of schizophrenia: a selective review. Ann Acad Med Singapore.

[7] Imamura Y, Nakane Y, Ohta Y, Kondo H (1999). Lifetime prevalence of schizophrenia among individuals prenatally exposed to atomic bomb radiation in Nagasaki City. Acta Psychiatr Scand.

[8] Meyer U, Feldon J (2010). Epidemiology-driven neurodevelopmental animal models of schizophrenia. Prog Neurobiol.

[9] Weinstock M (2008). The long-term behavioural consequences of prenatal stress. Neurosci Biobehav Rev.

[10] Seckl JR (2004). Prenatal glucocorticoids and long-term programming. Eur J Endocrinol.

[11] de Kloet ER, Sibug RM, Helmerhorst FM, Schmidt MV (2005). Stress, genes and the mechanism of programming the brain for later life. Neurosci Biobehav Rev.

[12] Beydoun H, Saftlas AF (2008). Physical and mental health outcomes of prenatal maternal stress in human and animal studies: a review of recent evidence. Paediatr Perinat Epidemiol.

[13] Lee PR, Brady DL, Shapiro RA (2007). Prenatal stress generates deficits in rat social behavior: reversal by oxytocin. Brain Res.

[14] Kinnunen AK, Koenig JI, Bilbe G (2003). Repeated variable prenatal stress alters pre- and postsynaptic gene expression in the rat frontal pole. J Neurochem.

[15] Koenig JI, Elmer GI, Shepard PD (2002). Stress during gestation produces alterations in adult rat behavior: relevance to schizophrenia. Soc Neurosci abs. 495.6.

[16] Koenig JI, Elmer GI, Shepard PD (2005). Prenatal exposure to a repeated variable stress paradigm elicits behavioral and neuroendocrinological changes in the adult offspring: potential relevance to schizophrenia. Behav Brain Res.

[17] Hayashi A, Nagaoka M, Yamada K (1998). Maternal stress induces synaptic loss and developmental disabilities of offspring. Int J Dev Neurosci.

[18] Lemaire V, Koehl M, Moal LM, Abrous DN (2000). Prenatal stress produces learning deficits associated with an inhibition of neurogenesis in the hippocampus. Proc Natl Acad Sci USA.

[19] Martínez-Téllez RI, Hernández-Torres E, Gamboa C, Flores G (2009). Prenatal stress alters spine density and dendritic length of nucleus accumbens and hippocampus neurons in rat offspring. Synapse.

[20] Van den Hove DL, Kenis G, Brass A (2012). Vulnerability versus resilience to prenatal stress in male and female rats; implications from gene expression profiles in the hippocampus and frontal cortex. Eur Neuropsychopharmacol.

[21] Mairesse J, Vercoutter-Edouart AS, Marrocco J (2012). Proteomic characterization in the hippocampus of prenatally stressed rats. J Proteomics.

[22] Lee HY, Joo J, Nah SS Changes in Dpysl2 expression are associated with prenatally stressed rat offspring and susceptibility to schizophrenia in humans. Int J Mol Med.

[23] Vogler BK, Pittler MH, Ernst E (1999). The efficacy of ginseng. A systematic review of randomised clinical trials. Eur J Clin Pharmacol.

[24] Dan B, Andrew G (1993). Chinese Herbal Medicine.

[25] Helms S (2004). Cancer prevention and therapeutics: Panax ginseng. Altern Med Rev.

[26] Park JD, Rhee DK, Lee YH (2005). Biological activities and chemistry of saponins from Panax ginseng C. A. Meyer. Phytochem Rev.

[27] Qi LW, Wang CZ, Yuan CS (2011). Isolation and analysis of ginseng: advances and challenges. Nat Prod Rep.

[28] Chen EY, Hui CL (2012). HT1001, a proprietary North American ginseng extract, improves working memory in schizophrenia: a double-blind, placebo-controlled study. Phytother Res.

[29] Dulawa SC, Holick KA, Gundersen B, Hen R (2004). Effects of chronic fluoxetine in animal models of anxiety and depression. Neuropsychopharmacology.

[30] Schroeder M, Sultany T, Weller A (2013). Prenatal stress effects on emotion regulation differ by genotype and sex in prepubertal rats. Dev Psychobiol.

[31] Axel B, Brigitte P, Helmut S (2003). Ketamin-induced changes in tar behavior: a possible animal model of schizophrenia. Prog Neuropsychopharmacol Biol Psychiatry.

[32] Joo J, Lee S, Nah SS (2013). Lasp1 is down-regulated in NMDA receptor antagonist-treated mice and implicated in human schizophrenia susceptibility. J Psychiatr Res.

[33] Goshima Y, Nakamura F, Strittmatter P, Strittmatter SM (1995). Collapsin-induced growth cone collapse mediated by an intracellular protein related to UNC-33. Nature.

[34] Attele AS, Wu JA, Yuan CS (1999). Ginseng pharmacology: multiple constituents and multiple actions. Biochem Pharmacol.

[35] Bahrke MS, Morgan WR (2000). Evaluation of the ergogenic properties of ginseng: an update. Sports Med.

[36] Radad K, Gille G, Liu L, Rausch WD (2006). Use of ginseng in medicine with emphasis on neurodegenerative disorders. J Pharmacol Sci.

[37] Liu CX, Xiao PG (1992). Recent advances on ginseng research in China. J Ethnopharmacol.

[38] Baek NI, Kim DS, Lee YH (1996). Ginsenoside Rh4, a genuine dammarane glycosidefrom Korean red ginseng. Planta Med.

[39] Christensen LP (2009). Ginsenosides chemistry, biosynthesis, analysis and potential health effects. Adv Food Nutr Res.

[40] Kim DH, Moon YS, Jung JS (2003). Effects of ginseng saponin administered intraperitoneally on the hypothalamo-pituitary-adrenal axis in mice. Neurosci Lett.

[41] Fugh-Berman A, Cott JM (1999). Dietary supplements and natural products as psychotherapeutic agents. Psychosom Med.

[42] Radad K, Gille G, Moldzio R (2004). Ginsenosides Rb1 and Rg1 effects on mesencephalic dopaminergic cells stressed with glutamate. Brain Res.

[43] Park EK, Choo MK, Oh JK (2004). Ginsenoside Rh2 reduces ischemic brain injury in rats. Biol Pharm Bull.

[44] Ji YC, Kim YB, Park SW (2005). Neuroprotective effect of ginseng total saponins in experimental traumatic brain injury. J Korean Med Sci.

[45] Van Kampen J, Robertson H, Hagg T, Drobitch R (2003). Neuroprotective actions of the ginseng extract G115 in two rodent models of Parkinson’s disease. Exp Neurol.

[46] Dang H, Chen Y, Liu X (2009). Antidepressant effects of ginseng total saponins in the forced swimming test and chronic mild stress models of depression. Prog Neuropsychopharmacol Biol Psychiatry.

[47] Cui J, Jiang L, Xiang H (2011). Ginsenoside Rb3 exerts antidepressant-like effects in several animal models. J Psychopharmacol.

[48] Shen L, Zhang J (2003). Ginsenoside Rg1 increases ischemia-induced cell proliferation and survival in the dentategyrus of adult gerbils. Neurosci Lett.

[49] Borrell J, Vela JM, Arevalo-Martin A (2002). Prenatal immune challenge disrupts sensorimotor gating in adult rats. Implications for theetiopathogenesis of schizophrenia. Neuropsychopharmacology.

[50] Zuckerman L, Rehavi M, Nachman R, Weiner I (2003). Immune activation during pregnancy in rats leads to a postpubertal emergence of disrupted latent inhibition, dopaminergic hyperfunction, and altered limbic morphology in the offspring: a novel neurodevelopmental model of schizophrenia. Neuropsychopharmacology.

[51] Palmer AA, Printz DJ, Butler PD (2004). Prenatal protein deprivation in rats induces changes in prepulse inhibition and NMDA receptor binding. Brain Res.

[52] Kelley ME, Gilbertson M, Mouton A, van Kammen DP (1992). Deterioration in premorbid functioning in schizophrenia: a developmental model of negative symptoms in drug-free patients. Am J Psychiatry.

[53] Moller P, Husby R (2000). The initial prodrome in schizophrenia: searching for naturalistic core dimensions of experience and behavior. Schizophr Bull.

[54] Cornblatt BA (2002). The New York high risk project to the Hillside recognition and prevention (RAP) program. Am J Med Genet.

[55] Weinstock M (2001). Alterations induced by gestational stress in brain morphology and behavior of the offspring. Prog Neurobiol.

[56] Arimura N, Menager C, Fukata Y, Kaibuchi K (2004). Role of CRMP-2 in neuronal polarity. J Neurobiol.

[57] Lin PC, Chan PM, Hall C, Manser E (2011). Collapsin response mediator proteins (CRMPs) are a new class of microtubule-associated protein (MAP) that selectively interacts with assembled microtubules via a taxol-sensitive binding interaction. J Biol Chem.

[58] Higurashi M, Iketani M, Takei K (2012). Localized role of CRMP1 and CRMP2 in neurite outgrowth and growth cone steering. Dev Neurobiol.

[59] Brittain JM, Piekarz AD, Wang Y (2009). An atypical role for collapsin response mediator protein 2 (CRMP-2) in neurotransmitter release via interaction with presynaptic voltage-gated calcium channels. J Biol Chem.

[60] Johnston-Wilson NL, Sims CD, Hofmann JP (2000). Disease-specific alterations in frontal cortex brain proteins in schizophrenia, bipolar disorder, and major depressive disorder. The Stanley Neuropathology Consortium. Mol Psychiatry.

[61] Cassereau J, Nicolas G, Lonchampt P (2013). Axonal regeneration is compromised in NFH-LacZ transgenic mice but not in NFH-GFP mice. Neuroscience.

